# A novel mutation in the *CDH1* gene in a Spanish family with hereditary diffuse gastric cancer

**DOI:** 10.1186/s40064-016-2852-7

**Published:** 2016-07-26

**Authors:** María López, Cristina Cervera-Acedo, Paula Santibáñez, Raquel Salazar, Jesús-Javier Sola, Elena Domínguez-Garrido

**Affiliations:** 1 Molecular Diagnostics Laboratory, Fundación Rioja Salud, Logroño, La Rioja Spain; 2 Department of Medical Oncology, Hospital San Pedro, Logroño, La Rioja Spain; 3 Department of Pathological Anatomy, Hospital San Pedro, Logroño, La Rioja Spain

**Keywords:** *CDH1*, Diffuse gastric cancer, Mutation, E-cadherin

## Abstract

Hereditary diffuse gastric cancer (HDGC) is an inherited form of diffuse type gastric cancer. Germline *CDH1* mutations have been identified in approximately 15–50 % of affected kindred that meet the clinical criteria for HDGC. If any of the criteria is met the individual is referred to genetic counseling and *CDH1* testing is offered. In this report we present the case of a Spanish family with HDGC harboring a novel *CDH1* mutation. A 47 year-old female with a diagnostic of gastric adenocarcinoma and some of her relatives were tested. Study of the entire *CDH1* gene, including intron–exon boundaries, by PCR and sequencing and immunohistochemical determination of the expression of E-cadherin were performed. A novel heterozygous deletion in exon 9 of *CDH1* gene (c.1220_1220delC, p.P407Qfs10), was found in the proband, one sister and a nephew. It generates a premature stop codon giving rise to a truncated protein that leads to a pathogenic variant. Expression of E-cadherin was absent or frankly reduced in the proband’s tumor but normal in tumor cells of great-uncle. After these results, the sister underwent prophylactic total gastrectomy, and the nephew is under annual endoscopic surveillance. Personal or familial history of diffuse gastric cancer, above all at young age, should encourage *CDH1* genetic testing. In this sense, the review of the criteria and the addition in the last guideline of the recommendation: “other families in which genetic testing may also be considered” broadens the number of individuals at risk detected. Since there are not reliable methods for early detection, DGC is usually diagnosed at an advanced stage and consequently associated with a poorer outcome. Thus, *CDH1* mutations detection contributes to an improvement in diagnosis and therapeutic intervention.

## Introduction

Gastric cancer (GC) is currently the fifth most common cancer being the third leading cause of cancer death worldwide, with nearly one million new diagnoses per year. More than three-fourths of those individuals die from the disease (Black et al. 2014; GLOBOCAN 2012). Most of GC cases are sporadic and hereditary cases account for only 1–3 % of GCs, this includes hereditary diffuse gastric cancer (HDGC) (Monahan and Hopkins 2016).

HDGC (OMIM #137215) is an autosomal dominant genetic predisposition cancer syndrome with high penetrance. Between 25 and 30 % of cases of HDGC are caused by mutations in *E*-*cadherin* gene (*CDH1*) (Hallowell et al. 2016; Hansford et al. 2015). This gene maps to chromosome 16q22.1, consists of 16 exons and encodes the cell-to-cell adhesion protein, E-cadherin (Masciari et al. 2007). To date, more than 180 different germline *CDH1* mutations have been identified in HDGC families in a diverse range of ethnic groups. Mutation carriers have a cumulative risk of GC at 80 years of 70 % for men and 56 % in women, together with a high probability of lobular breast cancer in females (Corso et al. 2014; Hallowell et al. 2016; Hansford et al. 2015). Germline *CDH1* mutations have been identified in approximately 15–50 % of affected kindreds that meet the clinical criteria for HDGC. The wide range of this estimate has to do with both, the background incidence of gastric cancer and the criteria used to define the syndrome. According to the new guidelines, defined in 2015, *CDH1* testing should be considered in patients who meet one of the following criteria (including first and second degree relatives): (1) 2 GC cases regardless of age, at least one confirmed diffuse gastric cancer (DGC); (2) one case of DGC before 40 years old or (3) personal or family history of DGC and LBC, one diagnosed before 50 years old. In addition, testing could be considered in families with: (1) Bilateral lobular breast cancer (LBC) or family history of 2 or more cases of LBC before 50 years old; (2) A personal of family history of cleft lip/palate in a patient with DGC or (3) in situ signet ring cells and/or pagetoid spread of signet ring cells (van der Post et al. 2015). Because DGC is often asymptomatic until in its advanced stages, the diagnosis is often delayed and, as a result, the prognosis is poor. Thus, according to current guidelines, prophylactic total gastrectomy (PTG) should be strongly advised in asymptomatic carriers of *CDH1* pathogenic mutations, since this is the only way to completely eradicate their risk of GC; furthermore, almost 100 % of performed gastrectomies revealed the presence of microscopic cancer foci The optimal timing for the surgery is under debate, although most procedures are performed between the ages of 20 and 30 years, and current guidelines advocate this (Monahan and Hopkins 2016, van der Post et al. 2015).

In this report, we describe the identification of a family in Spain, affected with HDGC, and carrying a novel germline truncating mutation in the *CDH1* gene (c.1220_1220delC, p.P407Qfs10) which presumably leads to a non-functional protein.

## Case description

### Patient

A 47 year-old female had been complaining of asthenia and a significant weight loss in the previous 2 months. She was smoker (about 20 cigarettes per day) and had no underlying diseases such as diabetes or hypertension. Test to detect *Helicobacter pylori* was negative. After gastroendoscopy and other explorations, such as computerized axial tomography scan, histological analysis of the tumour confirmed the diagnostic of gastric adenocarcinoma: diffuse adenocarcinoma with signet-ring cells. The proband died during the study.

**Fig. 1:**
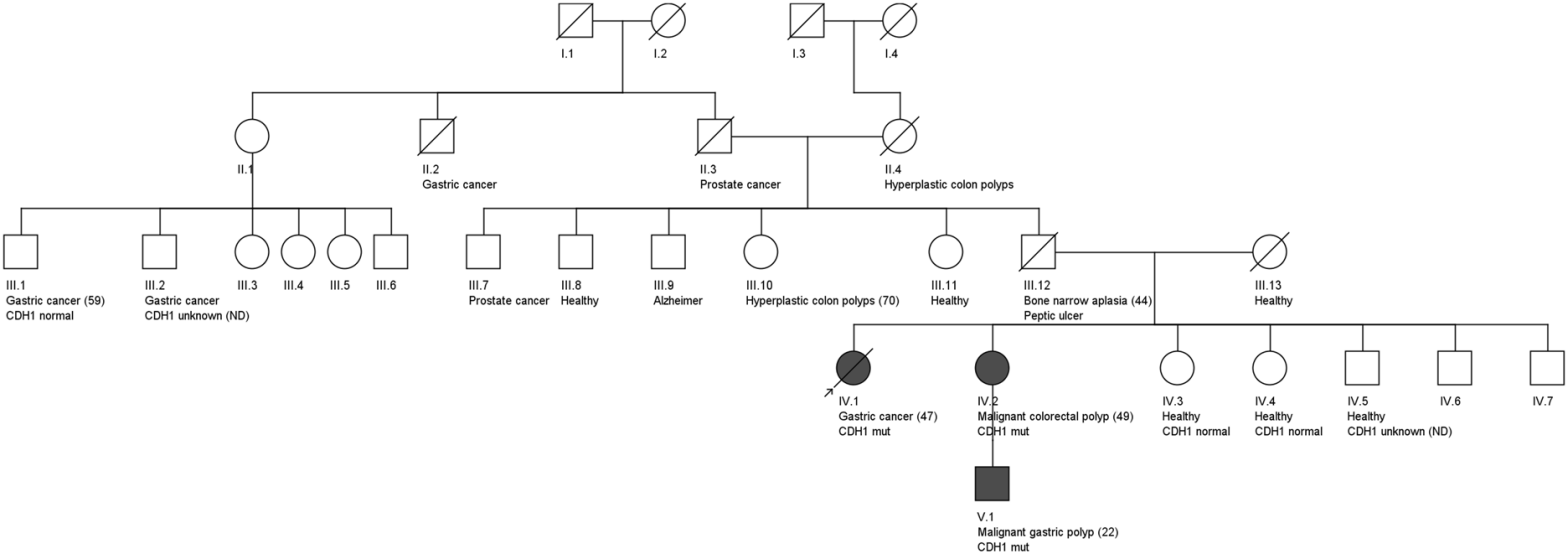
Family pedigree. *Shaded circles* and *squares* indicate the presence of the familiar mutation in *CDH1* gene

Some of her relatives presented previous history of malignant colorectal polyp (one sister), hyperplastic gastric polyp (a nephew) or gastric cancer (great-uncle) (Fig. 1). Thus, search for *CDH1* germline mutations was conducted in the patient and in other members of the family (three sisters, a great-uncle and a nephew of the proband), after genetic counseling and informed consent.

### Immunohistochemical analyses

In order to determine the expression level of the protein, paraffin tissue samples were subjected to immunohistochemical staining with monoclonal antibody against E-cadherin (clone 36B5, Leica Biosystems, UK) in an automated Bond system in combination with Bond Polymer Refine Detection (Leica Biosystems, UK) according to manufacturer instructions.

### CDH1 study

Genomic DNA extraction was carried out from peripheral blood by using QIAamp DNA Blood Mini Kit, and from paraffin embedded tissue (from proband’s gastric biopsy) with QIAamp DNA FFPE Tissue Kit; following manufacturer instructions. In the proband, RNA was also isolated from peripheral blood with QIAamp RNA Blood Mini Kit and from tissue using RNeasy FFPE Kit, and then stored at −80 °C.

The *CDH1* gene was studied by analyzing the entire coding sequence, including intron–exon boundaries, by PCR and sequencing. The presence of the mutation was confirmed at RNA level in the proband material.

Pathogenicity of the new variant was predicted by in silico analysis with bioinformatics tools such as sorting intolerant from tolerant (SIFT) and Mutation Taster. ExAC browser of Broad Institute, 1000 Genomes database and dbSNP138, as well as, the human gene mutation database (HGMD), Leiden open variation database (LOVD) and ClinVar databases were checked to assess the presence/absence of detected alterations in variations repositories.

### Identification of a novel mutation in the CDH1 gene

A novel heterozygous deletion in exon 9 of *CDH1* gene (NM_004360.4:c.1220_1220delC; NM_004360.4 (CDH1_i001): p.(Pro407Glnfs*10)), has been found in the proband and subsequently in other family members. This variant was not found in 100 healthy controls and it is not present in 1000G, ExAC and dbSNP, pointing out that this variant is not common in population. To the best of our knowledge this variant has not been previously described, and it is not included in ClinVar, HGMD or LOVD. Variant has been included in ClinVar database (SCV000266475). This mutation, generates a premature stop codon at position 407 giving rise to a truncated protein, that leads to a pathogenic variant (Corso et al. 2014). The presence of the mutation was corroborated both, at DNA and RNA level in peripheral blood. The mutation was not found in 100 control samples. The variant was considered pathogenic/disease causing by in silico predictors SIFT and Mutation Taster. Furthermore, this variation is considered to be pathogenic, according to American College of Medical Genetics and Genomics (ACMG) interpretation: null variant in a gene where loss of function is a known mechanism o disease, absent in population databases, protein length changing variant, co-segregation with disease in multiple affected family members in a gene definitively known to cause the disease, and patient’s phenotype highly specific for gene (Richards et al. 2015).

One of the sisters (subject IV-2), who was found a malignant colorectal polyp at the age of 49, presented also the deletion, and her son (nephew of the proband; (subject V-1)) harboured also the mutation and had been previously diagnosed with hyperplastic gastric polyp. Neither of the other relatives tested harboured the mutation and they were healthy, but for the great-uncle (subject III-1) who had been suffered indeterminate-type GC and did not present the mutation. There were other relatives which had been suffered hyperplastic colon polyps (subjects II-4; III-10), GC (subject II-2; III-2), and other kind of cancer (subjects II-3; III-7), but samples were not available for this study.

The sister, carrier of the mutation, and asymptomatic at the time of this study, underwent PTG. Her son (subject V-1), refused the procedure and is under annual endoscopic surveillance (recently, hyperplastic gastric polyps has been detected).

It was not possible to establish the origin of this mutation with the data collected. It should be necessary to test more relatives in order to determine in which point its origin is.

### Expression of E-cadherin in the tumour tissue

**Fig. 2:**
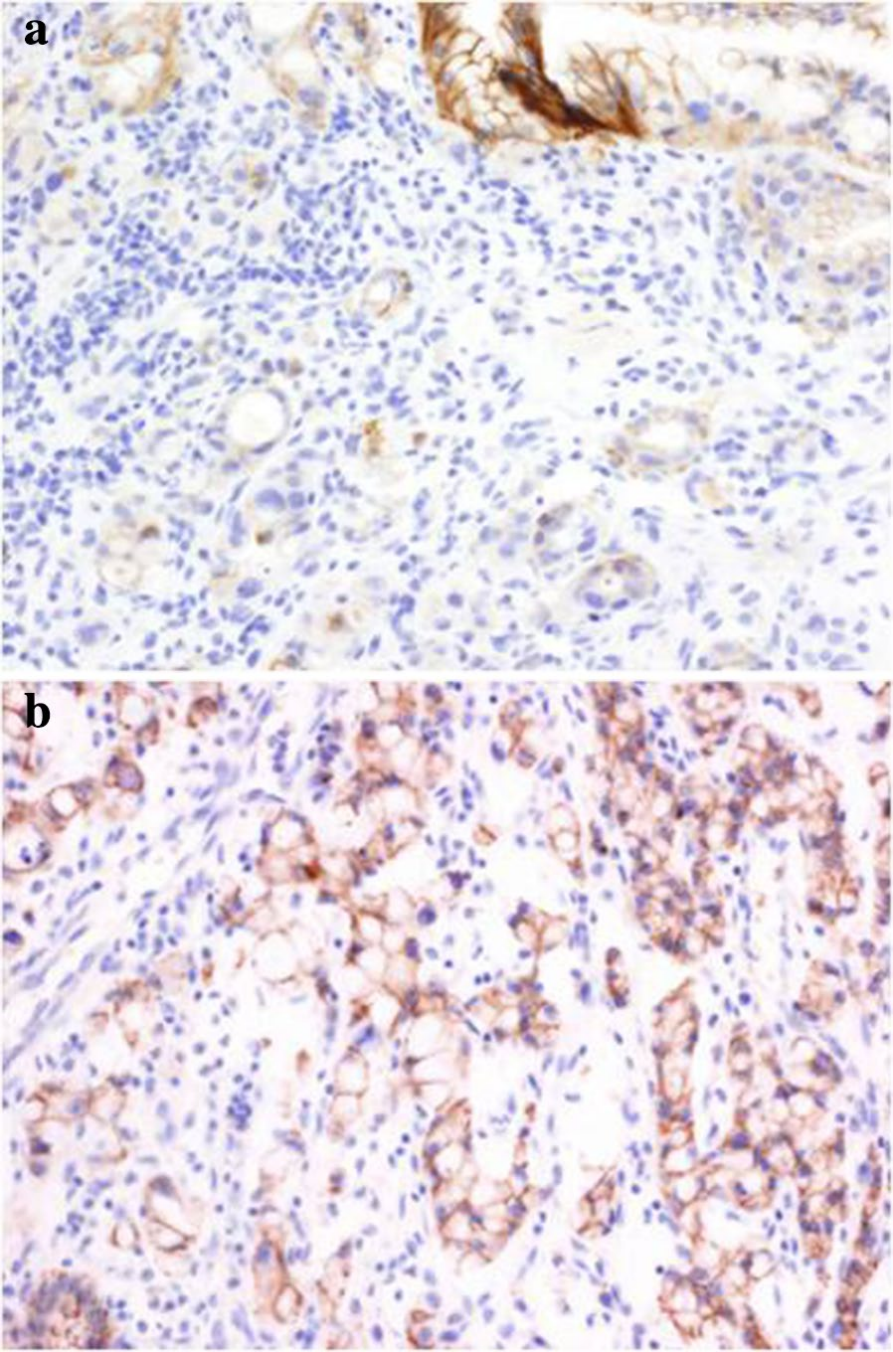
Immunohistochemical expression of E-cadherin (IHQx400). **a** Tumor cells of proband gastric biopsy. **b** Gastric tumor cells of great-uncle

In the tumor cells of proband’s gastric biopsy, the immunohistochemical expression of E-cadherin was absent or frankly reduced. In contrast, normal continuous membranous staining for E-cadherin was evidenced in gastric tumor cells of great-uncle (Fig. 2).

## Discussion and evaluation

In this paper the identification of a novel mutation in the *CDH1* gene in a Spanish family diagnosed with GC is described. For HDGC, there are established criteria to determine whether an individual is at risk of and requires genetic testing for the condition. In the last revision, the first and second criteria were merged in a new criterion: “Two or more GC cases regardless of age, at least one confirmed DGC”, in first-degree and second-degree relatives, covering now families for whom detailed pathology is incomplete (Fitzgerald et al. 2010; van der Post et al. 2015).

In this way, despite our patient did not fulfill the strict criteria for HDGC, due to the young age of diagnosis, the histopathological analysis of the tumour (diffuse adenocarcinoma with signet-ring cells), and with an E-cadherin expression absent, she was tested for *CDH1* mutations, and once she was found to have a pathogenic mutation, genetic testing was offered to some of her relatives. Our finding reinforces the addition in the last guideline of the recommendation: “other families in which genetic testing may also be considered” that includes a point that our patient had met: “in situ signet ring cells and/or pagetoid spread of signet ring cells” (van der Post et al. 2015). According to our findings, personal or familial history of DGC, above all at young age, should encourage *CDH1* genetic testing, even without fulfilling HDGC criteria. The detection of well known pathogenic mutations in sporadic cases of GC that do not meet HDGC criteria (Garziera et al. 2013), supports the importance of searching for *CDH1* mutations in this type of cancer, not only to better understand the molecular basis underlying the disease, but also to improve genetic testing and therefore the clinical management of patients and families at risk.

Pathology reports, preferably by an expert GC pathologist, are essential. The pathology of HDGC is unique but requires a high level of expertise in order to maximize recognition of specific findings. Because of the detection of in situ signet ring cells and/or pagetoid spread of signet ring cells in the stomach is rarely, if ever, seen in sporadic cases, and in these cases genetic testing should be considered.

First degree relatives of GC patients are known to have twofold to threefold increased risk of GC. This shows the importance of informing family members about positive mutation results, facilitating a faster testing, diagnosis and treatment (Garziera et al. 2013; Onitilo et al. 2013). The optimal age to screen individuals from affected families is unclear. Rare cases of DGC have been reported in affected families before the age of 18, but the overall risk of cancer before age 20 is very low (<1 %) (Kaurah et al. 2007; Pharoah et al. 2001). The risk rises to 4 percent by age 30 without prophylactic surgery. Most groups agree that consideration of genetic testing can begin at the age of informed consent (16 or 18 years of age depending on the geographic place of residence) (Blair et al. 2006; van der Post et al. 2015). However, decisions as to the age at which to institute testing should also take into account the earliest age of cancer onset in the individual family.

PTG is now strongly recommended for asymptomatic *CDH1* mutation carriers. Total gastrectomy for these patients completely eliminates their risk of GC and is truly prophylactic in terms of preventing their death from invasive GC. The current consensus is that the procedure should be discussed and offered to pathogenic *CDH1* carriers in early adulthood, generally between ages 20 and 30 and should be carefully considered at an age >75. Family phenotype, especially age of onset of clinical cancer in probands, should be taken into account. Some suggest consideration of total gastrectomy in *CDH1* mutation carriers at an age 5 years younger than the youngest family member who developed gastric cancer (Cisco et al. 2008). In *CDH1* positive patients who deny or want to delay the PTG endoscopic surveillance should be considered.

According to recent studies, there is a significant difference between asymptomatic and symptomatic patients who undergo prophylactic total gastrectomy. These studies demonstrated that asymptomatic patients were all cured after the surgery whereas a high percentage of symptomatic ones had tumour recurrence or metastasis, and 60 % of them died within 2 years (Chen et al. 2013; Corso et al. 2014). In addition, data from over 100 gastrectomies for HDGC have high-lighted the majority already contain a tiny focus of signet-ring carcinoma or the preinvasive lesions.

The mutation found in our family: (NM_004360.4:c.1220_1220delC; NM_004360.4(CDH1_i001): p.(Pro407Glnfs*10) generates an early stop codon in the protein leading to a truncated protein and thus, pathogenic. To date, over 180 different germline *CDH1* mutations have been identified; the majority are pathogenic mutations but a number of variants of uncertain significance (VUS) have been described (Hansford et al. 2015; van der Post et al. 2015). The majority has been single nucleotide substitutions leading to non-synonymous changes (splice site or truncating mutations); less commonly, there are insertions or deletions of several base-pairs leading to frameshifts with protein truncation. Approximately 5 % of familial cases are due to large deletions involving multiple exons of the gene (Oliveira et al. 2009; Yamada et al. 2014). All germline mutations are evenly distributed along the gene and lead to functional haploinsufficiency of E-cadherin. *CDH1* is a tumor suppressor gene, and therefore a somatic second hit is required for initiation of tumor formation. The trigger and molecular mechanism by which the second allele of E-cadherin is inactivated appears to be diverse, and includes promoter hypermethylation, mutation, and loss of heterozygosity. The end result is loss of expression of the cell adhesion molecule E-cadherin.

Currently, there are not reliable methods for early detection, and GC patients have often a poor prognosis since it is often detected at advanced states, more aggressive and difficult to treat, being considered not curable (Black et al. 2014; Garziera et al. 2013; Onitilo et al. 2013). Annual endoscopic surveillance is recommended but direct visualization with endoscopy tends to detect lesions late in the disease process and multiple random endoscopic samples often returns false negatives. Better surveillance methods could reduce morbidity by picking up target lesions earlier such that they are amenable to endoscopic therapies.

## Conclusions

In conclusion, although the HDGC is a rare disease and its incidence is low, due to the high pathogenicity and penetrance importance should be attached to it. The lack of a sensitive screening test for HDGC makes its early diagnosis challenging. In this sense, the establishment of well defined criteria for the detection of families at risk is essential. The identification of *CDH1* mutation may provide valuable information for genetic counseling, as well as comprehensive management and confirmatory diagnosis of HDGC and GC risk reduction for the as-yet unaffected family members.

## Abbreviations

GC: gastric cancer
HDGC: hereditary diffuse gastric cancer
DGC: diffuse gastric cancer
*CDH1*: *E*-*cadherin* gene
LBC: lobular breast cancer
SIFT: sorting intolerant from tolerant
HGMD: human gene mutation database
LOVD: Leiden open variation database
ACMG: American College of Medical Genetics and Genomics
PTG: prophylactic total gastrectomy
VUS: variants of uncertain significance
